# Overexpression of Mitochondrial Ferritin Enhances Blood–Brain Barrier Integrity following Ischemic Stroke in Mice by Maintaining Iron Homeostasis in Endothelial Cells

**DOI:** 10.3390/antiox11071257

**Published:** 2022-06-26

**Authors:** Peina Wang, Qianqian Ren, Mengtong Shi, Yuanyuan Liu, Huiyuan Bai, Yan-Zhong Chang

**Affiliations:** 1 Laboratory of Molecular Iron Metabolism, Key Laboratory of Animal Physiology, Biochemistry and Molecular Biology of Hebei Province, Ministry of Education Key Laboratory of Molecular and Cellular Biology, College of Life Science, Hebei Normal University, Shijiazhuang 050024, China; hbsdwpn@163.com (P.W.); 15732153064@163.com (Q.R.); shimengtong2022@163.com (M.S.); yuanyuan-liu@outlook.com (Y.L.); huiyuanbai7@163.com (H.B.); 2 Department of Histology and Embryology, College of Basic Medical Sciences, Hebei Medical University, Shijiazhuang 050017, China

**Keywords:** blood–brain barrier, ischemic stroke, mitochondrial ferritin, iron, endothelial cells

## Abstract

Blood–brain barrier (BBB) breakdown, a characteristic feature of ischemic stroke, contributes to poor patient outcomes. Brain microvascular endothelial cells (BMVECs) are a key component of the BBB and dysfunction or death of these cells following cerebral ischemia reperfusion (I/R) injury can disrupt the BBB, leading to leukocyte infiltration, brain edema and intracerebral hemorrhage. We previously demonstrated that mitochondrial ferritin (FtMt) can alleviate I/R-induced neuronal ferroptosis by inhibiting inflammation-regulated iron deposition. However, whether FtMt is involved in BBB disruption during cerebral I/R is still unknown. In the present study, we found that FtMt expression in BMVECs is upregulated after I/R and overexpression of FtMt attenuates I/R-induced BBB disruption. Mechanistically, we found that FtMt prevents tight junction loss and apoptosis by inhibiting iron dysregulation and reactive oxygen species (ROS) accumulation in I/R-treated BMVECs. Chelating excess iron with deferoxamine alleviates apoptosis in the brain endothelial cell line bEnd.3 under oxygen glucose deprivation followed by reoxygenation (OGD/R) insult. In summary, our data identify a previously unexplored effect for FtMt in the BBB and provide evidence that iron-mediated oxidative stress in BMVECs is an early cause of BMVECs damage and BBB breakdown in ischemic stroke.

## 1. Introduction

Stroke, of which approximately 71% are ischemic strokes, is the second leading cause of death and a major cause of long-term disability worldwide [1]. Currently, the only approved treatment for ischemic stroke is thrombolysis. Due to the narrow therapeutic time window and the high risk of intracerebral bleeding complications, only a few patients can benefit from such therapy [2]. In addition, blood reperfusion after thrombolysis treatment may induce severe secondary damage to the brain, known as ischemia/reperfusion (I/R) injury, which results in irreversible neurological injury [3,4,5]. Although the mechanism of I/R injury remains ill-defined, increasing evidence suggests that blood–brain barrier (BBB) disruption plays a key role in the pathological process of ischemic stroke [6].

The BBB is composed of endothelial cells, astrocytic end feet, pericytes and basement membranes, which together modulate the entry of substances or cells circulating in the blood into the brain to maintain a steady environment for normal brain function [7,8]. Structurally, tight junctions (TJs), composed of transmembrane proteins, including zonula occludens (ZOs), claudins and occludin, between brain endothelial cells play a crucial role in maintaining the integrity of the BBB [9,10]. I/R injury usually leads to mitochondrial dysfunction, oxidative stress, inflammatory responses and apoptosis, with a disruption of TJ integrity, resulting in BBB leakage and brain damage [11]. Importantly, the increased permeability of the BBB can promote the progress and enlargement of the injury by increasing the risk of hemorrhage, causing cerebral edema, and aggravating the inflammatory response [12,13]. Clinically, the degree of BBB damage correlates with low survival rates in ischemic stroke patients [2,14]. While pharmacological treatment that ameliorates BBB damage can effectively improve the outcomes of patients with ischemic stroke, as well as stroke model mice [15,16]. Endothelial cells play a key role in maintaining vascular function and integrity and are the most important component of the BBB [17]. Endothelial cell dysfunction or death has been shown to contribute to BBB disruption and vascular injury in cerebral I/R [18,19]. However, the molecular mechanism of endothelial cell death and the subsequent BBB disruption remain to be elucidated.

Mitochondrial ferritin (FtMt), a mitochondrial iron storage protein, plays a key role in regulating cellular iron homeostasis and maintaining the cellular redox balance [20,21]. FtMt can catalyze the conversion of ferrous iron to the ferric form for storage in the FtMt spherical shell. Its expression is tissue-specific, with high levels in cell types of high metabolic activity, such as the testes and brain, with undetectable expression in the main iron storage tissues, including the liver and spleen [22]. Our previous work, and that of others, have shown that I/R injury causes brain iron dysregulation and iron content is increased in ischemic brains [23,24]. The excess free iron can promote free radical accumulation in neuronal cells by catalyzing the Fenton reaction. We recently demonstrated that FtMt is mainly expressed in neurons, rather than glial cells, and that the protein can alleviate I/R-induced brain damage by inhibiting iron-induced oxidative stress and ferroptosis in neurons [21,25]. In addition, we observed an enhanced activation of microglia and upregulation of pro-inflammatory genes in FtMt knockout mice after I/R, indicating that FtMt can protect the brain against the I/R-induced inflammatory response [25]. However, whether cerebral microvascular endothelial cells (BMVECs) express FtMt and whether FtMt can protect the integrity of the BBB during cerebral I/R have not yet been investigated.

In the present study, we used FtMt-overexpressing mice and a brain endothelial cell line, bEnd.3, as well as mouse models of middle cerebral artery occlusion (MCAO) stroke and oxygen–glucose deprivation followed by reoxygenation (OGD/R) to investigate the role and mechanisms of FtMt in BBB disruption during cerebral I/R. We found that FtMt was upregulated in BMVECs after I/R and overexpression of FtMt alleviated I/R-induced BBB disruption. FtMt attenuated the loss of TJ proteins and degeneration of endothelial cells during ischemic stroke. Mechanistically, our results provide evidence that FtMt prevents I/R-induced iron dysregulation in brain endothelial cells, resulting in decreased production and accumulation of ROS, thus markedly inhibiting endothelial cell apoptosis and BBB disruption. The understanding of the role of FtMt in maintaining BBB integrity and function may represent a target for the development of safe and effective therapeutic approaches to protect the BBB integrity in ischemic stroke.

## 2. Materials and Methods

### 2.1. Animals

C57BL/6J wild-type male mice and FtMt-overexpressing male mice (12 weeks old) were used in the present study. We generated and genotyped FtMt-overexpressing mice as previously reported [26]. All animals were housed under controlled conditions (temperature 22 °C ± 2 °C, humidity 50 ± 5%, and 12 h/12 h light/dark cycle) and were provided standard rodent diet and water ad libitum. Animal experiments were carried out in accordance with the National Institutes of Health Guide for the Care and Use of Laboratory Animals and were approved by the Animal Care and Use Committee of the Hebei Science and Technical Bureau in China (protocol code 2021LLSC034).

### 2.2. MCAO Model Mice

MCAO surgery was performed in adult mice to induce ischemic stroke as previously reported [25]. In brief, mice were weighed and then anesthetized with an intraperitoneal injection of chloral hydrate (3.5 mg/kg). The common carotid artery (CCA), external carotid artery (ECA) and internal carotid artery (ICA) were carefully separated. A nylon monofilament (602234PK10Re, Doccol Corp., Sharon, MA, USA) was inserted into the beginning of the middle cerebral artery (MCA) through the ECA and ICA until the ipsilateral blood flow decreased to less than 30% of baseline, as monitored by laser Doppler flowmetry (Perimed, Järfälla, Sweden). After 1 h of ischemia, the suture was removed to commence reperfusion. Body temperature was maintained at 37 °C during the entire procedure with a heating blanket. Animals were allowed access to food and water ad libitum after MCAO surgery. Mice that had excessive bleeding during the surgery or experienced cerebral hemorrhage, or when the filament was removed before reperfusion, were excluded from the study.

### 2.3. Measurement of Evans Blue (EB) Extravasation

BBB permeability was investigated by measuring EB dye extravasation as previously reported [27]. EB dye (2% in saline, 4 mL/kg) was injected into ischemic mice through the caudal vein 22 h after reperfusion and allowed to circulate for 2 h. Next, the mice were anesthetized and perfused transcardially with saline. To quantify EB leakage, the two hemispheres of the brain were dissected and weighed prior to further analysis. The samples were homogenized in 2 mL saline and 1.5 mL 60% trichloroacetic acid to extract the EB dye. After vortexing for 2 min, the mixture was centrifuged for 30 min at 1000× *g* and the supernatant was collected. The absorption of the supernatant was measured at 610 nm using a microplate reader. The EB dye content was compared with a standard curve and is expressed as µg/g brain tissue.

### 2.4. Isolation of Brain Microvascular Endothelial Cells

I/R mice were transcardially perfused with cold saline and the brain was quickly isolated. The contralateral hemisphere and the ipsilateral hemisphere were separated and homogenized with cold buffer containing 10 mM HEPES, 4 mM KCl, 147 mM NaCl, 2.5 mM CaCl_2_, 1 mM MgSO_4_·7H_2_O, 1 mM NaH_2_PO_4_·2H_2_O, 1 mM sodium pyruvate and 10 mM glucose. Next, dextran (Solarbio) was added to the mixture to a final concentration of 16%. The samples were centrifuged at 7200× *g* for 15 min at 4 °C and the supernatant was discarded. The pellet was resuspended in the buffer and then the suspension was centrifuged at 3500× *g* for 10 min at 4 °C. The supernatant was removed and the pellet, the purified BMVECs, was used for further analysis.

### 2.5. Western Blot Analysis

Western blot was performed by a standard protocol as we previously described [28]. After electrophoresis, the proteins were transferred onto nitrocellulose membranes and blocked with 5% non-fat milk in Tris-buffered saline for 90 min. The membranes were then incubated with primary antibodies overnight at 4 °C. The following primary antibodies were used: anti-β-actin (1:10,000) and anti-transferrin receptor 1 (TfR1; 1:2000) were obtained from Sigma-Aldrich (St. Louis, MO, USA) (#A5441, #SAB4200398); anti-ferroportin1 (FPN1; 1:5000) and anti-divalent metal transporter 1 (DMT1±IRE; 1:5000) were obtained from Alpha Diagnostic International (San Antonio, TX, USA) (#MTP11-S, #NRAMP21-S, #NRAMP23-S); anti-4-hydroxynonenal (4-HNE), anti-mitochondrial ferritin (1:5000), anti-ferritin light chain (FtL; 1:10,000) and anti-ferritin heavy chain (FtH; 1:10,000) were obtained from Abcam (#ab46545, #ab66111, #ab109373, #ab183781); anti-caspase3 (1:5000), anti-Bcl-2 (1:2000) and anti-Bax (1:2000) were obtained from Cell Signaling Technology (Danvers, MA, USA) (#14220, #3498, #2772); anti-ZO-1 (1:2000), anti-occludin (1:2000) and anti-claudin-5 (1:2000) were obtained from Thermo Fisher (Waltham, MA, USA) (#PA5-85256, #40-4700, #34-1600). After washing, the membranes were incubated with secondary antibodies. Finally, the signals were detected using the enhanced chemiluminescence (ECL) method and quantified by transmittance densitometry using ImageJ software.

### 2.6. Immunofluorescence (IF)

Double-IF was performed as previously described [29]. Briefly, 15-μm-thick, frozen brain slices were washed three times with phosphate-buffered saline (PBS) and then antigen retrieval was performed in a microwave oven for 10 min in 0.01 M citrate buffer (pH 6.0). After blocking for 60 min in goat serum, the slices were incubated with primary antibodies (CD31, BD Bioscience, #562939; ZO-1, Thermo Fisher #PA5-85256; occludin, Thermo Fisher #40-4700; claudin-5, Thermo Fisher #34-1600) overnight at 4 °C. The slices were washed three times within PBS and then the corresponding secondary antibodies (DyLight 488, Abbkine; DyLight 549, Abbkine) were provided at 37 °C for 60 min. Finally, the slices were stained with DAPI (Thermo Fisher #62248) for 4 min. After washing and mounting, the sections were analyzed with an Olympus FV3000 confocal laser scanning microscope.

### 2.7. Cell Culture and Deferoxamine (DFO) Treatment

Immortalized mouse brain endothelial cells of the bEnd.3 cell line were cultured in DMEM (Dulbecco’s modified Eagle’s medium) supplemented with fetal calf serum (10%, *vol*/*vol*), glucose (4.5 mg/mL), penicillin (100 U/mL) and streptomycin (100 µg/mL). bEnd.3 cells were maintained in a 37 °C humidified incubator with 5% CO_2_ and 95% air. For chelation treatment, the cells were incubated with 5 mM DFO (Sigma-Aldrich, St. Louis, MS, USA) at the beginning of reperfusion.

### 2.8. Oxygen and Glucose Deprivation and Reperfusion

The OGD/R model was performed to mimic ischemia reperfusion in vitro. The medium was completely removed from bEnd.3 cells, after which the cells were washed twice in PBS and glucose-free DMEM was added. The plates were placed in a hypoxic chamber with 1% O_2_/5% CO_2_/94% N_2_ at 37 °C. OGD was carried out for 9 h. Reoxygenation was initiated by adding complete medium, and the cells were incubated under normal conditions for another 2 h. Control cells were incubated with complete medium for 9 h and replaced with complete medium under normal conditions in a manner identical to that for OGD/R cells.

### 2.9. Measurement of Cell Viability

Cell viability was measured by the MTT assay. After OGD/R insult, the culture medium was discarded and then the cells were incubated with 0.5 mg/mL MTT at 37 °C in a 5% CO_2_ incubator for 4 h. The supernatants were removed and the formazan was dissolved using dimethyl sulfoxide. The absorbance of the solution was measured at 570 nm by a microplate reader.

### 2.10. Assessment of Apoptosis by Flow Cytometry

Apoptosis was measured by flow cytometry with a FITC-Annexin V apoptosis detection kit (#C1062L, Beyotime, Shanghai, China) according to the manufacturer’s instructions. In brief, bEnd.3 cells were harvested and stained with Annexin V and propidium iodide (PI) for 10 min at 37 °C after OGD/R treatment. The cells were then centrifuged at 1000× *g* for 5 min at room temperature and washed twice with PBS. The percentage of apoptotic cells was analyzed using a flow cytometer (CytoFLEX, Beckman Coulter, Brea, CA, USA).

### 2.11. Measurement of Lipid ROS and Mitochondrial ROS

The production of lipid ROS and mitochondrial ROS in cells after OGD/R treatment were assessed with C11-BODIPY (581/591) and MitoSOX (#D3861, #M36008, Invitrogen, Waltham, MA, USA), respectively. The cells were harvested and washed twice with Hanks balance salt solution (HBSS). The cells were then resuspended in 500 µL HBSS containing 2 μM C11-BODIPY (581/591) or 5 µM MitoSOX for 10 min at 37 °C. After washing three times with HBSS, the cells were analyzed by flow cytometry.

### 2.12. Measurement of Malondialdehyde (MDA) and Superoxide Dismutases (SODs)

MDA and total SOD levels in bEnd.3 cells after OGD/R insult were assessed by commercial kits (#S0131M, #S0101S) from the Beyotime Institute of Biotechnology (Shanghai, China) according to the manufacturer’s directions. In the MDA assay, MDA in cell samples can react with thiobarbituric acid (TBA) under high temperature and acidic conditions to produce a red-colored MDA-TBA adduct. The MDA concentrations were measured by the absorbance of the product at 531 nm. The SOD activity in cells was assessed using the WST-8 colorimetric method. The absorbance of the reactive product at 560 nm was recorded with a microplate reader. The concentration of total protein in samples was quantified by the BCA kit.

### 2.13. Statistical Analysis

Data were analyzed using GraphPad Prism-7 and SPSS 16.0. All data are presented as the mean ± SEM. Two-tailed Student’s *t*-test was used for two group comparisons and one-way ANOVA with Tukey’s post hoc test used for multi-group comparisons. A value of *p* < 0.05 was considered statistically significant.

## 3. Results

### 3.1. Overexpression of FtMt Attenuates I/R-Induced BBB Disruption

**Figure 1:**
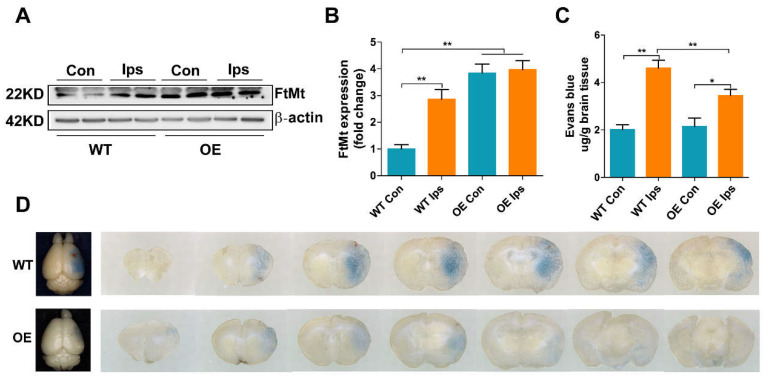
Overexpression of FtMt attenuates I/R-induced BBB disruption. (**A**) Ischemic stroke was induced in wild-type mice (WT) and FtMt-overexpressing mice (OE) by MCAO for 60 min with a subsequent 24 h reperfusion. The expression of FtMt in the contralateral hemisphere (Con) and ipsilateral hemisphere (Ips) was measured. (**B**) Quantification of FtMt levels. The data are expressed relative to the mean value in the WT Con group (*n* = 4). (**C**) Quantification of Evans blue dye extravasation in WT or OE mice after I/R (*n* = 4). (**D**) Representative images of Evans blue dye extravasation in WT or OE mice after I/R. The results are presented as the mean ± SEM. * *p* < 0.05, ** *p* < 0.01.

To examine whether FtMt may contribute to maintaining the integrity of the BBB during cerebral I/R, we measured the expression of FtMt in BMVECs and the effect of FtMt on I/R-induced BBB disruption. We observed that FtMt was indeed expressed in BMVECs and the level of FtMt was significantly upregulated in ipsilateral (Ips) BMVECs compared with the contralateral (Con) samples during ischemic stroke (Figure 1A,B). Moreover, the expression of FtMt in BMVECs of FtMt-overexpressing mice (OE) was markedly increased compared with the wild-type mice (WT) Con group, including the WT Ips group, while there was no difference between the OE Con group and OE Ips group (Figure 1A,B). Next, we evaluated the effect of FtMt on I/R-induced BBB disruption by performing EB extravasation. As shown in Figure 1C,D, while apparent increases in EB extravasation were observed in both WT and OE mice after I/R treatment, the BBB integrity of the FtMt-overexpressing mice was better than that of the WT mice, indicating that FtMt aids in the preservation of BBB integrity after MCAO.

### 3.2. Overexpression of FtMt Increases Tight Junctional Integrity in Cerebral I/R

**Figure 2:**
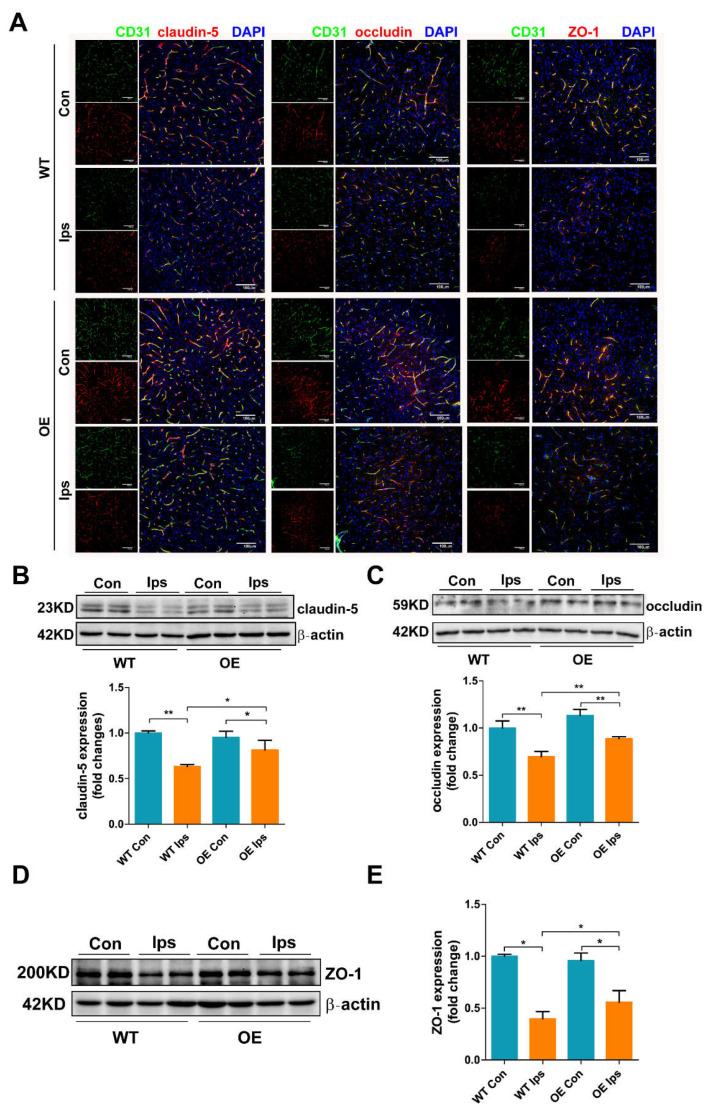
Overexpression of FtMt increases tight junctional integrity in cerebral I/R. (**A**) Immunofluorescence staining for claudin-5, occludin, ZO-1 and CD31 with DAPI counterstaining in the cortex of WT and OE mice after I/R (*n* = 3). Scale bar: 100 µm. Western blot analysis of (**B**) claudin-5, (**C**) occludin and (**D**,**E**) ZO-1 from ischemic brains. The data are expressed relative to the mean value in the WT Con group (*n* = 4). The results are presented as the mean ± SEM. * *p* < 0.05, ** *p* < 0.01.

The TJs between endothelial cells serve to restrict substances in the blood from entering the brain. To verify whether FtMt overexpression increases the BBB integrity by attenuating the degradation of junction-related proteins that occurs after ischemic stroke, we immunofluorescence-stained the tight junctional molecules claudin-5, occludin and ZO-1, along with the endothelial marker CD31. After I/R injury, the intensities of claudin-5, occludin and ZO-1 were diminished in the vessels of the Ips hemisphere of WT mice (Figure 2A). In contrast, the expression of these tight junctional proteins was significantly increased in the OE Ips group compared with the WT Ips group. Western blot analysis further confirmed the immunofluorescence results; the expression of claudin-5 (Figure 2B), occludin (Figure 2C) and ZO-1 (Figure 2D,E) were significantly decreased in the WT Ips group; however, all of these proteins were markedly less affected with FtMt overexpression. These results demonstrate that FtMt overexpression protects BBB integrity via increased endothelial tight junction expression, thus decreasing vascular leakage after cerebral I/R.

### 3.3. Overexpression of FtMt Abrogates Endothelial Cell Apoptosis after Cerebral I/R

**Figure 3:**
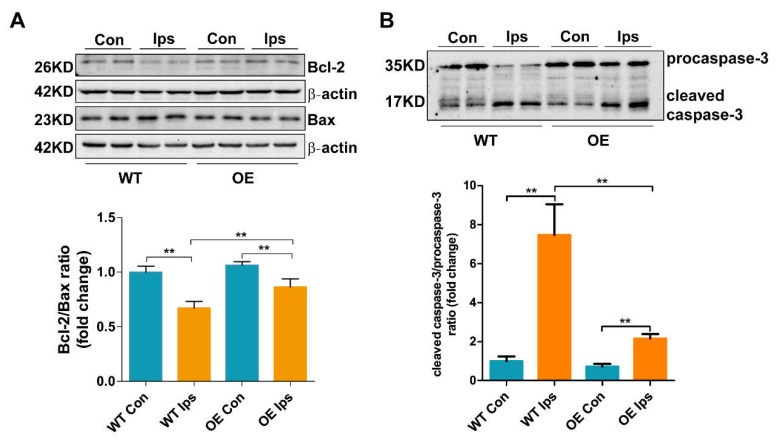
Overexpression of FtMt abrogates endothelial cell apoptosis after cerebral I/R. Western blot and densitometric analyses of (**A**) the ratio of Bcl-2 to Bax, (**B**) the ratio of cleaved caspase-3 to procaspase-3 (*n* = 4). The data are expressed relative to the mean value in the WT Con group (*n* = 4). The results are presented as the mean ± SEM. ** *p* < 0.01.

As the main cell type of the BBB, BMVEC survival is critical for the formation of TJs and the maintenance of BBB integrity. In order to determine the effects of FtMt on endothelial cell apoptosis after ischemic stroke, we assessed the expression of biomarkers for apoptosis in BMVECs of the WT and OE mice after I/R. As shown in Figure 3A,B, the Bcl2/Bax ratio was decreased, whereas the cleaved caspase-3/procaspase-3 ratio was markedly increased in the WT Ips group. FtMt overexpression significantly attenuated the I/R-induced decrease in the Bcl2/Bax ratio and increase in the caspase-3/procaspase-3 ratio in BMVECs, indicating that the overexpression of FtMt was protective against I/R-induced BMVEC apoptosis.

### 3.4. Overexpression of FtMt Rescues I/R-Induced Iron Dysregulation and Oxidative Stress in BMVECs

**Figure 4:**
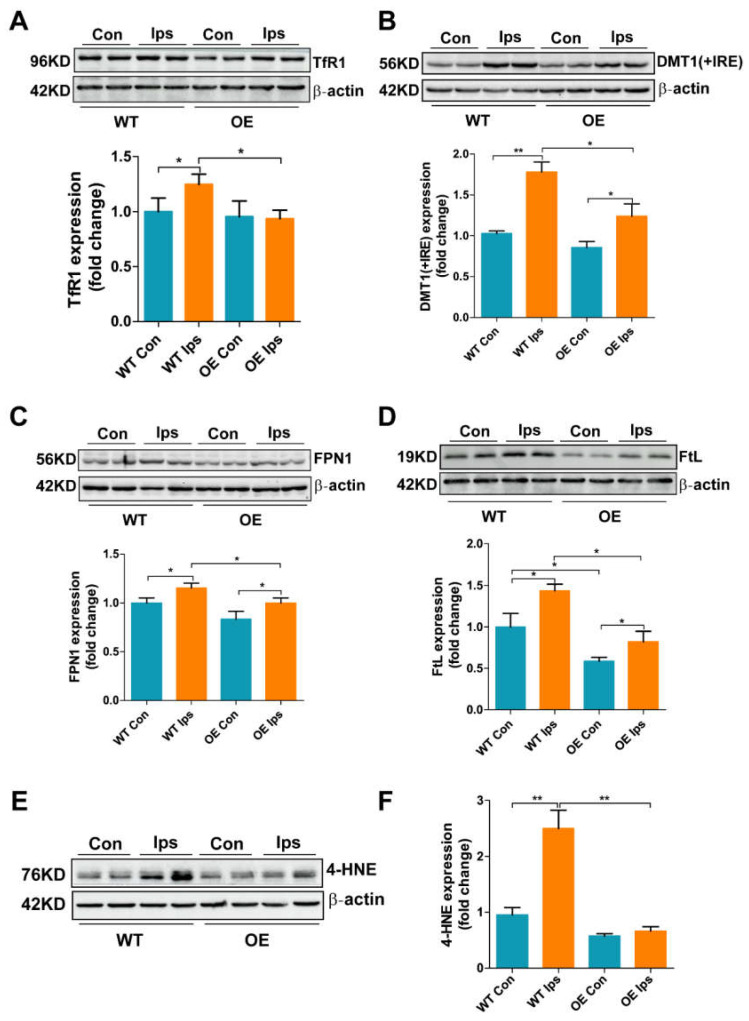
Overexpression of FtMt rescues I/R-induced iron dysregulation and oxidative stress in BMVECs. Western blot and densitometric analyses of (**A**) TfR1, (**B**) DMT1(+IRE), (**C**) FPN1, (**D**) FtL and (**E**,**F**) 4-HNE. The results are normalized to the β-actin levels and the data are expressed relative to the mean value in the WT Con group (*n* = 4). The results are presented as the mean ± SEM. * *p* < 0.05, ** *p* < 0.01.

Substantial evidence has indicated that ROS accumulation is a key factor in BMVEC apoptosis in I/R [30]. Our previous studies demonstrated that brain iron accumulation and the subsequent generation of ROS are key factors mediating neuronal cell death [24,25]. However, whether iron dysregulation is also a cause of BMVEC death in I/R remains unclear. To further elucidate the mechanism by which FtMt suppresses I/R-induced BMVWC apoptosis, we evaluated the expression of iron metabolism-related proteins and indicators of oxidative stress. The levels of iron uptake proteins TfR1 (transferrin receptor 1) and DMT1(+IRE) (divalent metal transporter 1) were significantly increased in the WT Ips group compared with the WT Con, indicating that I/R stimulates endothelial cells to transport more iron from the blood into the cytosol. Overexpression of FtMt inhibited the increases in TfR1 and DMT1(+IRE) levels (Figure 4A,B). In addition, we observed increased levels of FPN1 (ferroportin 1), the only cellular iron exporter, in BMVWCs after I/R, with the level in the OE Ips group significantly decreased compared to that in the WT Ips group (Figure 4C). The expression of FtL, an indicator of cytosolic iron levels, was markedly increased, while this increase was inhibited in FtMt-overexpressing mice. These data confirm that I/R causes iron dysregulation and accumulation in BMVWCs and that this is prevented by FtMt (Figure 4D). Upregulation of FPN1 may be the result of feedback regulation on iron overload in BMVWCs after I/R. Moreover, the levels of 4-HNE (4-hydroxynonenal), an end product of lipid oxidation and a biomarker of oxidative stress, in BMVECs was increased after I/R, with FtMt overexpression leading to a significant decrease in this indicator (Figure 4E,F). Taken together, these results indicate that FtMt inhibits I/R-induced iron overload in BMVECs, which can further block ROS generation and the subsequent apoptosis.

### 3.5. OGD/R Induces Iron Dysregulation and Oxidative Stress in bEnd.3 Cells

**Figure 5:**
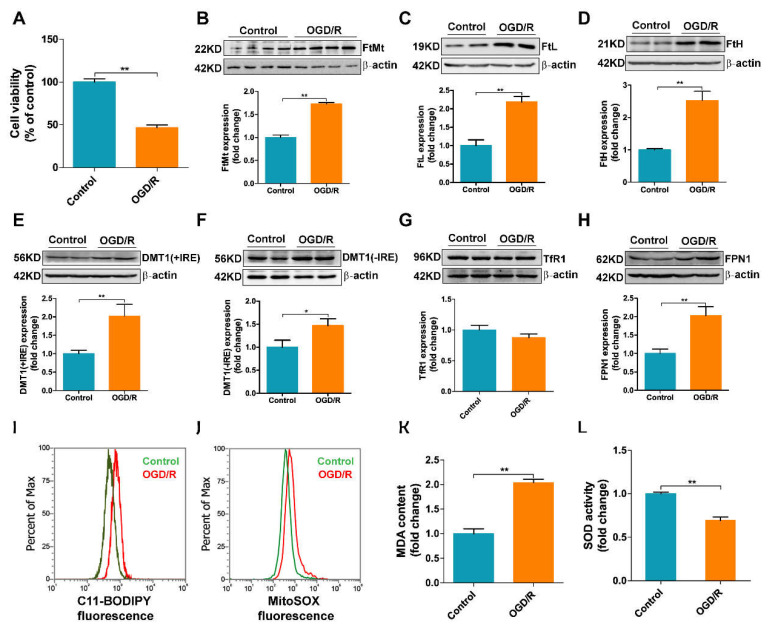
OGD/R induces iron dysregulation and oxidative stress in bEnd.3 cells. bEnd.3 cells were subjected to OGD for 9 h and reperfusion for 2 h. (**A**) Cell viability was determined using the MTT assay (*n* = 6). The levels of (**B**) FtMt, (**C**) FtL, (**D**) FtH, (**E**) DMT1(+IRE), (**F**) DMT1(-IRE), (**G**) TfR1 and (**H**) FPN1 were analyzed by western blot and the results were normalized to the β-actin levels. The data are expressed relative to the mean value in the control group (*n* = 4). (**I**) Lipid ROS and (**J**) mitochondrial ROS production was assessed in bEnd.3 cells after OGD/R insult by flow cytometry using C11-BODIPY and MitoSOX, respectively; representative data from one of three experiments are shown. (**K**) MDA and (**L**) SOD activity were analyzed by commercial assays and the data are expressed relative to the mean value in the control group (*n* = 3). The results are presented as the mean ± SEM. * *p* < 0.05, ** *p* < 0.01.

To validate the role of iron in I/R-induced endothelial cell injury, we further investigated the expression of iron-related proteins in bEnd.3 cells after OGD/R insult. As shown in Figure 5A, after the cells were subjected to 9 h of OGD followed by 2 h of reoxygenation, the cell viability was decreased by about 50%, indicating that the in vitro I/R model was successfully established. Consistent with the in vivo results, the expression of FtMt was significantly upregulated after OGD/R insult, and the levels of FtL and FtH were also increased (Figure 5B–D). Thus, OGD/R appears to lead to iron accumulation in bEnd.3 cells. Moreover, the levels of DMT1(+IRE) and DMT1(-IRE) were both increased compared with the control group, which may, at least in part, explain the increased iron uptake in bEnd.3 cells after OGD/R insult (Figure 5E,F). We did not observe any significant increase in TfR1 expression in the OGD/R group like that in the mice model (Figure 5G), which is likely due to differences between culture plate and whole animal. FPN1 levels also increased after OGD/R insult (Figure 5H). In addition, flow cytometry results show that the formation of lipid ROS and mitochondrial ROS in bEnd.3 cells after OGD/R treatment were markedly increased (Figure 5I,J). Analysis of the MDA content in cells also consistently demonstrated the same result (Figure 5K). The activity of SOD, a free-radical scavenging enzyme, in the OGD/R group was significantly decreased (Figure 5L). These results demonstrate that iron dysregulation and ROS accumulation also occur in bEnd.3 cells in an in vitro I/R model.

### 3.6. OGD/R Induces Tight Junction Loss and Apoptosis in bEnd.3 Cells

**Figure 6:**
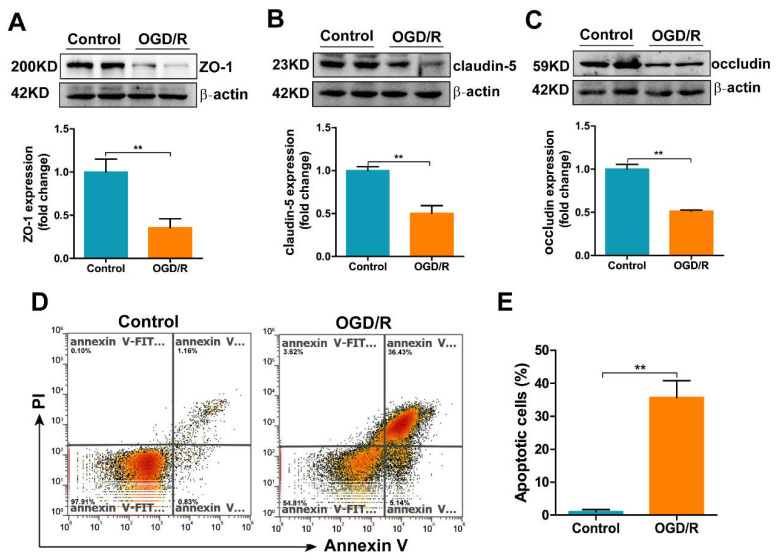
OGD/R induces tight junction loss and apoptosis in bEnd.3 cells. Western blot and densitometric analyses of (**A**) ZO-1, (**B**) claudin-5 and (**C**) occludin in OGD/R-treated bEnd.3 cells. The results are normalized to the β-actin levels and the data are expressed relative to the mean value in the control group (*n* = 4). (**D**) Apoptosis was measured by flow cytometry; representative data from one of three experiments are shown. (**E**) Quantification of the percentage of apoptotic cells in OGD/R-treated bEnd.3 cells. The results are presented as the mean ± SEM. ** *p* < 0.01.

We next assessed the expression of TJ proteins, including ZO-1, claudin-5 and occludin, as well as the apoptosis levels in OGD/R-treated bEnd.3 cells. As shown in Figure 6A–C, the TJ proteins exhibited decreased expression in the OGD/R group. To assess whether the tight junction loss may be a consequence of apoptosis, we measured apoptosis in bEnd.3 cells after OGD/R insult. The percentage of apoptotic cells rose to nearly 35% after OGD/R treatment (Figure 6D,E).

### 3.7. DFO Treatment Attenuates OGD/R-Induced Oxidative Damage in bEnd.3 Cells

**Figure 7:**
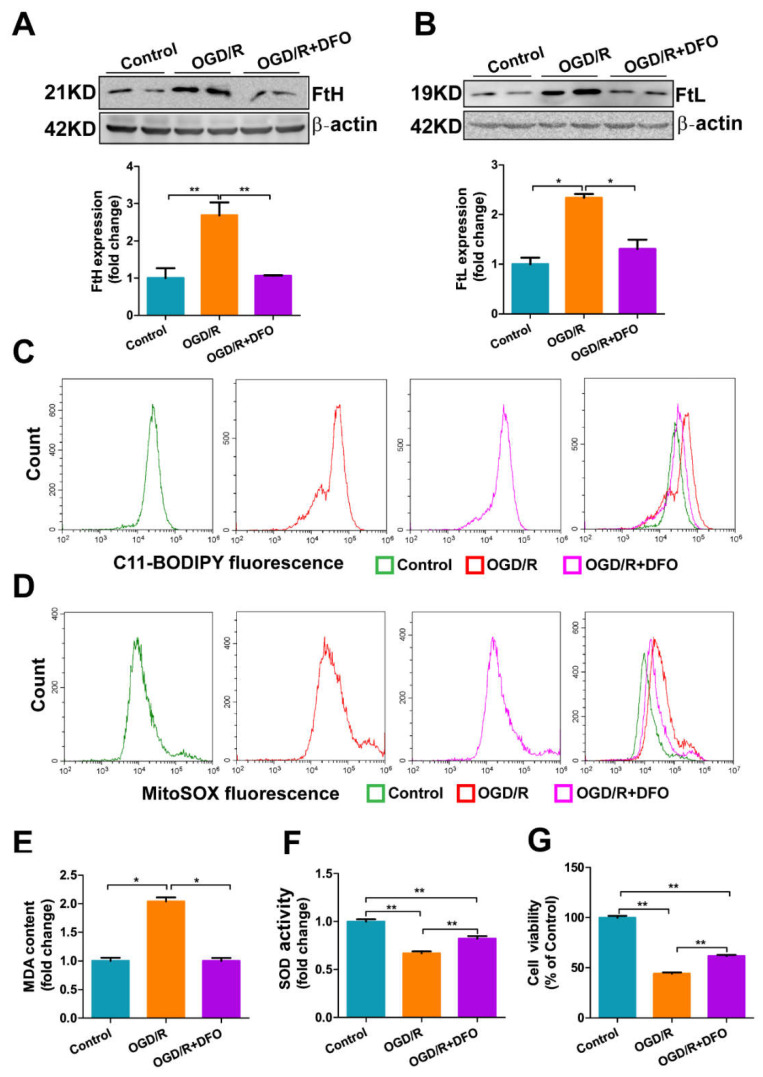
DFO treatment attenuates OGD/R-induced oxidative damage in bEnd.3 cells. bEnd.3 cells were subjected to OGD treatment for 9 h and then reoxygenation in the presence of 5 µM DFO for 2 h. Western blot and densitometric analyses of (**A**) FtH and (**B**) FtL. The results are normalized to the β-actin levels and the data are expressed relative to the mean value in the control group (*n* = 4). (**C**) Lipid ROS and (**D**) mitochondrial ROS levels were assessed in OGD/R-treated bEnd.3 cells with or without added DFO by flow cytometry using C11-BODIPY and MitoSOX, respectively; representative data from one of three experiments are shown. (**E**) MDA and (**F**) SOD activity were analyzed by commercial assays and the data are expressed relative to the mean value in the control group (*n* = 3). (**G**) Cell viability was determined using the MTT assay (*n* = 6). The results are presented as the mean ± SEM. * *p* < 0.05, ** *p* < 0.01.

To validate that increased iron was a major cause of the damage to bEnd.3 cells induced by OGD/R insult, we applied the iron chelator DFO to the OGD/R-treated bEnd.3 cells. The levels of FtH and FtL decreased almost to the levels in the control group in the OGD/R-treated bEnd.3 cells when DFO was included during reoxygenation, indicating that the sequestration of iron was able to prevent OGD/R-induced iron accumulation (Figure 7A,B). Moreover, DFO attenuated ROS generation in OGD/R-treated bEnd.3 cells. The lipid ROS levels (Figure 7C), mitochondrial ROS levels (Figure 7D) and MDA content (Figure 7E) were significantly decreased, while the SOD content was markedly increased (Figure 7F) in the OGD/R+DFO group compared to the OGD/R group. As a result, DFO treatment significantly alleviated OGD/R-induced cell damage in bEnd.3 cells; the cell viability of OGD/R-treated bEnd.3 cells was increased when DFO was included during reoxygenation (Figure 7G). Taken together, these results demonstrate that iron overload is a key event in bEnd.3 cell injury under OGD/R insult, which is consistent with our in vivo data.

## 4. Discussion

Iron is an essential element for cell survival; many biochemical processes, such as DNA synthesis and oxygen transport, require iron [31]. The brain, a tissue with high metabolic activity and oxygen consumption, has a greater iron content than most organs [32]. The function of enzymes involved in cellular energy metabolism, synthesis of several neurotransmitters and the development of dendritic connections are all iron-dependent, so iron is vital for normal brain function and activity [33]. Importantly, as a transition metal, excess iron can catalyze hydroxyl radical production through the Fenton reaction to cause oxidative stress in neuronal cells [34]. Therefore, brain iron homeostasis is tightly regulated and iron dysregulation can lead to severe pathological damage in the nervous system, including cerebral I/R [35]. Previous studies have shown that poor clinical outcome correlates with elevated serum iron storage in stroke patients [35,36]. The transport of iron from the blood into the brain has to cross the BBB, after which the iron can be further taken up by neuronal cells. Our and others’ previous data indicate that the endothelial cells, as key component of the BBB, control the transport of iron into the brain [37,38]. In addition, we reported that iron homeostasis is disrupted in neuronal cells, including dysregulation of iron metabolism-related proteins and increased iron content, in the ischemic brain. However, as the gateway of iron transport across the BBB, brain microvascular endothelial cells may also experience iron dysregulation and iron-dependent oxidative stress, however this has not been explored so far. In the current study, we detected the effects of I/R on BMVEC iron metabolism in vivo and in vitro. We provided evidence that iron uptake proteins, including TfR1 and DMT1, were significantly upregulated in BMVECs after I/R (Figure 4A,B). Consequently, the iron content of I/R-treated BMVECs increased, leading to a stimulation of FtL expression (Figure 4D and Figure 5C). The increased expression of FPN1, the iron exporter, may be stimulated by high cellular iron content and may therefore contribute to the transport of iron into the brain (Figure 4C and Figure 5H). Excess iron in BMVECs exacerbated the generation of ROS and ultimately led to apoptosis after I/R (Figure 4E,F and Figure 5I–L). Iron chelation was able to reverse the OGD/R-induced endothelial cell injury (Figure 7). More importantly, we verified that FtMt can protect BMVECs from I/R-induced damage by restoring iron homeostasis.

Our previous studies demonstrated that FtMt prevents neuronal cells from oxidative stress injury in iron-related neurological diseases, including Alzheimer’s disease and Parkinson’s disease [28]. Recently, we found that FtMt was upregulated in the penumbra of the I/R brain and that FtMt can alleviate I/R-induced neuron ferroptosis by inhibiting the inflammatory response and iron overload [25]. In the current study, we extended this line of research to the role of FtMt in BMVECs and BBB integrity in the pathogenesis of I/R. We provide evidence that FtMt is not only expressed in neurons but also in BMVECs (Figure 1A,B). Overexpression of FtMt attenuated I/R-induced iron overload in BMVECs as well as the subsequent BMVEC apoptosis and BBB disruption (Figure 1, Figure 2, Figure 3 and Figure 4). Our data provide a new perspective for exploring the protective role of FtMt in ischemic stroke. On the one hand, FtMt in BMVECs improved BBB integrity that can inhibit excess iron transport into the brain through the injured endothelial cells or the damaged TJ. On the other hand, FtMt in neurons can improve antioxidative capacity of neurons and reduce neurological injury in I/R. However, why glial cells do not express FtMt, and whether FtMt impacts the communication between neurons/BMVECs and glial cells, still need to be elucidated in the future.

The integrity of the BBB is vital to the homeostasis of the central nervous system after ischemic stroke [17]. BBB disruption results in extravasation of blood cells, macromolecules and extracellular fluid into the brain. Infiltrated leukocytes can then release cytotoxic molecules, including nitric oxide, inflammatory cytokines and proteases, into the brain, which contributes to neuronal cell injury [39]. Therefore, elucidating the mechanisms that control BBB permeability in ischemic stroke is vitally important to minimize or prevent permanent brain injury. In this study, we demonstrated that endothelial cell apoptosis and the ensuing BBB disruption can be attributed, at least in part, to dysregulated iron homeostasis in endothelial cells. In addition, the ability of FtMt to reverse these effects in BMVECs may make the protein a potential target to protect against BBB dysfunction in I/R or other nerve system diseases.

During ischemic stroke, the depletion of oxygen and nutrients in the ischemic region of the brain triggers a cascade of detrimental processes leading to ROS generation, neuro inflammation and neuronal cell death. Therefore, the mechanisms of I/R injury are multifaceted and include oxidative stress, inflammation and excitotoxicity, among others [11]. Although numerous animal studies and clinical studies have been performed with the aim of identifying new treatment strategies for I/R, most of the findings to date have failed to translate to the clinic [40]. Thus, it remains essential to further elucidate the mechanisms of cerebral I/R and identify more effective treatments for I/R-induced brain injury. Endothelial cell death and destruction of BBB integrity and function are characteristic, early stage features of cerebral I/R injury but also the triggers that exacerbate downstream inflammatory responses and oxidative stress in neuronal cells. Therefore, we posit that treating BBB disruption in I/R injury is necessary to improve patient outcomes. Here, we have demonstrated that inhibiting iron overload in BMVECs by iron chelation or regulating iron metabolism-related proteins is beneficial to decreasing I/R-induced BBB disruption. It may provide new insight for the development of new treatments for ischemic stroke.

## 5. Conclusions

In the present study, we have demonstrated that FtMt is expressed in BMVECs and overexpression of FtMt reduces cerebral I/R-induced BBB disruption by inhibiting iron dysregulation and preventing BMVEC apoptosis.

## Data Availability

All of the data is contained within the article.
